# Can impoverished family nurture rich sons any more? The effect of household income on intergenerational transmission of education: Evidence from China

**DOI:** 10.3389/fpsyg.2023.1116217

**Published:** 2023-02-02

**Authors:** Xiaofan Li, Weiwei Xie, Lu Wang, Tingting Zou

**Affiliations:** ^1^Law and Business School, Wuhan Institute of Technology, Wuhan, China; ^2^Research Institute for Eco-civilization, Chinese Academy of Social Sciences, Beijing, China; ^3^Wuhan University of Engineering Science and Technology, Wuhan, China

**Keywords:** intergenerational transmission of education, household income, transmission matrix, intermediary effect, China

## Abstract

Family investment in education is an important variable influencing the educational attainment of children. Family investment in education is influenced by family income, and the increase in family income gap will aggravate the inequity of education and enhance the degree of intergenerational transmission of education. But the above theories need to be further tested in reality. This paper uses the 2018 China Family Panel Studies (CFPS) to verify the role of Chinese family income on intergenerational transmission of education through the education transition matrix and the mediating effect model, and examines the effect of college expansion policy on the mediating effect of family income on intergenerational transmission of education. The results show that: (1) The education level of parents has obvious transmissibility to the education level of children. The solidification rate of intergenerational transmission of education between parents and children is 25.72%, the upward mobility rate is 60.58% and the downward mobility rate is 13.70%. (2) The mediating effect model shows that the total effect of the parents’ education level on children’s education level is 0.279 and the direct effect is 0.272, and the family income plays a mediating effect in the intergenerational transmission of education, and the mediating degree reaches about 2.6%. (3) The expansion of higher education provides more opportunities for children of society, especially lower-middle-income families, to receive higher education, which weakens the mediating effect of family income in the intergenerational transmission of education. The findings of this paper provide support for policymakers to increase public investment in education.

## 1. Introduction

Since the implementation of the reform and opening-up policy in 1978, China has experienced rapid economic growth, but at the same time, the income gap among residents has been widening. One solution to the problem of how to narrow the income gap among residents is to promote equalization of education. Education, as a key production factor of human capital (An, 2005), plays an important role in the future development of individuals as well as in enhancing family income (Becker and Nigel, 1986). Educational functionalism argues that higher levels of education can help educated people acquire higher skills, thus enhancing intergenerational class mobility (Schneewind, 2015; Gul et al., 2020); conflict theory, on the other hand, believes that education is mainly a tool for social class reproduction and an intermediary medium for parents to pass social resources to their offspring (Bercovitch et al., 2009). In recent years, with the continuous expansion of education in China, the education level of the residents has been significantly improved, but there is also a gradual phenomenon of class solidification (Tadesse et al., 2022), and “it is difficult to produce noble children from a poor family” has been hotly debated in the society. This phenomenon reflects the importance people attach to social class solidification, and also reflects people’s concern about the intergenerational transmission of education. Intergenerational transmission of education is a form of intergenerational transmission, which means that the education level of parents has an impact on the education level of their children. Stronger intergenerational transmission of education means lower intergenerational mobility, and children of highly educated parents also have high education, which further contributes to class solidification and thus educational inequality; while weaker intergenerational transmission of education means stronger intergenerational mobility. When the intergenerational transmission of education is weaker, education will be more equal, the view of “reading changes fate” will be more recognized, and class mobility will be stronger.

There has been very extensive research on the intergenerational transmission effect of education, and most scholars believe that the intergenerational transmission effect of education exists (Sewell and Hauser, 1993; Chevalier et al., 2003). The educational level of parents can effectively explain the educational level of their children, and when parents have a high level of education, they are more likely to raise children with a high level of education (Haveman and Wolfe, 1995; Yang and Wan, 2015; Huo and Golley, 2022), and the educational level of children during their growth process depends to a large extent on the educational level of their parents (Kwenda et al., 2015). Treiman (1997) found that the number of years of education of parents had a positive effect on the number of years of education of the offspring, as shown by the fact that when the number of years of education of the parent increased by 1 year, the number of years of education of his children increased by half a year. Some studies have shown that the intergenerational transmission of educational attainment from parents to their offspring is strong in China (Li and Zhou, 2018; Dong et al., 2020), and that parents’ educational attainment has a greater impact on their children’s academic achievement; the longer the number of years of education of parents, the longer the number of years of education of their children (Qiu and Xiao, 2011; Wang et al., 2016; Fang and Feng, 2018). Based on CGSS data, Li and Huang (2020) conducted an empirical study on the current situation of intergenerational mobility in education in China using the probability transition matrix and intergenerational elasticity, and found that the educational attainment of Chinese parents positively contributes to the educational attainment of their children, with significant intergenerational transferability of education. Hiroshi and Li (2008) also concluded that parents’ education level has a highly significant positive effect on the education level of their offspring. Li (2003) found that children of parents with education level of high school and above will have relatively 2.3 years more years of education relative to parents with education level of high school and below; children of fathers with education level of junior high school and above will have 1 year more years of education compared to fathers with education level of illiterate/semi-literate. Sun and Yan (2015) found that parents’ education level positively influenced their children’s education level; specifically, children of fathers with high education levels were more likely to receive higher education, and mothers’ education level had a greater impact on their children’s education in junior high and high school (Zhou and Cheng, 2016).

Family income is a factor that directly affects the intergenerational transmission of education (Hanushek, 1986; Daviskean, 2005). Haveman and Wolfe (1995) found a significant effect of family income on the educational attainment of offspring. Brooks-Gunn and Duncan (1997) selected family socioeconomic status as a core variable and found that parental education and family socioeconomic status all had a significant effect on children’s educational attainment, with children from high-income families being more likely to receive higher education. Compared to parents who are not well educated, parents who are well educated can obtain higher social status and family income, and they pay more attention to their children’s education and are willing and able to invest more money and other resources to give their children more educational advantages (Yao et al., 2006; Ma and Wang, 2015; Wu and Huang, 2016; Wiedner and Schaeffer, 2020). With the development of China’s economy and society, the influence of family income and family conditions on individuals’ educational attainment gradually increased (Pang et al., 2013; Wang and Shi, 2014; Liu et al., 2015; Li and Zheng, 2017; Zou and Ma, 2019). As China’s social stratification intensified after 1992, the influence of family class background began to emerge, with higher-income families having more resources, an advantage that was reflected in the educational opportunities of the next generation (Wei et al., 2019). Using data from a national sample survey, Li (2003) found that household economic status has an increasing effect on children’s education, and that annual household income at age 14 has a significant effect on educational attainment for those in rural areas and for female students, but not for those in urban areas and for male students. A more precise study of her showed that children of fathers earning more than $2,000 per month had a higher chance of enrolling in undergraduate or professional education (Li, 2010). Hiroshi and Li (2008) analyzed regional differences in the intergenerational transmission of education and found that children of landowning or wealthy peasant families received relatively higher levels of education.

Relevant studies have shown that in the case of unequal distribution of educational resources, the influence of family income on individual educational attainment gradually increases (Huang, 2013), usually manifesting in the lower educational attainment of rural children compared to urban children, with significant differences between urban and rural areas. Despite the disparity in students’ family conditions and innate abilities, the schooling process should provide the fairest possible opportunity for each child. Then, in response to the phenomenon of educational inequality, how to effectively and rationally achieve resource allocation and promote educational equity is the focus of future research. This study investigates the effect of family income on intergenerational transmission of education using China Family Panel Studies (CFPS) data, and on this basis, we explore whether the policy of college expansion can attenuate this effect and provide a reference to alleviate the problem of unequal access to educational resources for disadvantaged families in China.

## 2. Data and method

### 2.1. Data source and description

The data are from China Family Panel Studies (CFPS), funded by Peking University and the National Natural Science Foundation of China. The CFPS is maintained by the Institute of Social Science Survey of Peking University. CFPS reflects social, economic, demographic, educational, and health changes in China by tracking and collecting data at the individual, household, and community levels. The CFPS is a nationwide, large-scale, multidisciplinary social tracking survey project that focuses on the economic and non-economic well-being of Chinese residents, as well as many research topics including economic activities, educational outcomes, family relationships and family dynamics, population migration, health, etc. The CFPS uses implicitly stratified, systematic probability sampling proportional to population size, and targets household households in 25 provinces of China and the sample of all household members in the family households. In the empirical analysis part of the study, this paper uses the household member relationship database, household economic database, child proxy database, and individual self-response database from the CFPS2018. Considering the intergenerational transmission of education as the influence of the educational level of the paternal members in a household on the educational level of their offspring, this paper selects the data of adults who have completed high school from the household member relationship pool to create an observation sample with the offspring as the basis.

The core variables in this paper include household income, and educational attainment of the parent and offspring. (1) Household income. In this paper, the net income of all households is selected as the measure of household income. (2) Education level. The educational level of parents is expressed by the highest education of father and mother as the educational level of parents, and the educational level of children is measured by the highest education of children. For the eight categories of “illiterate/semi-literate, elementary school, junior high school, high school (junior college/technical school/vocational high school), college, bachelor’s degree, master’s degree, and doctorate,” the corresponding years of education are 1, 6, 9, 12, 15, 16, 19, and 22 years, respectively. (3) The control variables selected in this article include intelligence level, health status, gender of children, number of family members, urban and rural population classification, and province of affiliation. The descriptive statistics of the variables selected in the article are shown in Table 1.

**Table 1:** Descriptive statistics of the sample.

Variable	Label	Obs	Mean	Std. Dev.	Min	Max
Parent_edu	Highest education of parents	5,039	2.86	1.09	1.00	7.00
ParenteduY	The maximum length of schooling of parents	5,039	8.32	3.57	1.00	19.00
Child_edu	Highest education of child	5,039	3.75	1.27	1.00	8.00
ChildeduY	The maximum length of schooling of child	5,039	10.95	3.57	1.00	22.00
Family size	Number of family members	5,039	4.36	1.92	1.00	16.00
Fincome	Family net income	5,039	9.14	16.12	0.00	506.50
Urban	Urban and rural classification based on China National Bureau of Statistics	5,012	0.44	0.50	0.00	1.00
Province	Chinese provinces	5,030	39.74	15.38	11.00	65.00
Health	level of health	3,622	5.47	1.28	1.00	7.00
Intelligence	Intelligence level	4,196	4.92	1.40	1.00	7.00

Table 1 demonstrates the mean, standard deviation, minimum and maximum values of the main variables in this paper, from which it can be seen that the average years of education of the offspring are 10.95 years, which basically reaches the high school level, and the years of education of the parents is 8.32 years. It indicates that with the socio-economic development, China pays more and more attention to education, the education level of the offspring has significant increase compared to their parents’ generation, and the offspring are getting more and more opportunities to receive education. As far as the data of other control variables are concerned, among the total sample of children, the percentage of males is 54.02% and the percentage of females is 45.98%, and in terms of urban–rural classification, the percentage of rural population is 56.48% and the percentage of urban population is 43.52%.

### 2.2. Method

(1) Switching matrix. The transition matrix, also known as the transfer probability matrix, is mainly used to study the mobility of income, the transition matrix provides us with the possibility to observe the flow of economic output at any point and is complementary to the intergenerational regression coefficient and the correlation coefficient. For the income dimension, the transition matrix can be measured using different income classes, in the general sense of a double random matrix of the following form (Cao and Liu, 2018).


(1) $$P\left(x,y\right)=p_{ij}\left(x,y\right)\in R_{+}{}^{m\times m}$$

Where $p_{ij}\left(x,y\right)$ represents the probability of an individual shifting from income level *i* in period *t* to income level *j* in period *t +*1. *m* is the number of rankings from lowest to highest by income level, which can be set by the analyst at any level as needed. All element values of this matrix are probabilities, so it takes values between 0 and 1; it is a doubly stochastic matrix, where larger elements on its main diagonal mean that individuals who were at a certain income level in the previous period are more likely to remain at the same income level in the current period, and therefore have less intergenerational income mobility (Formby et al., 2004). Similar to the income mobility matrix, the education transition matrix divides both the educational level of the parent and the educational level of the offspring into levels, with each row indicating the educational level of the offspring, each column indicating the educational level of the parent, and the numbers intersecting the rows and columns indicating the probability of their offspring entering each stage of education when the educational level of the parent is at that level (Li, 2018). Based on the intergenerational income conversion matrix established by Zhou and Zhang (2015), Ji and Liang (2020), and Xu and Mei (2021), this study introduces it into the intergenerational education conversion matrix study and constructs the following conversion matrix.


….(2) $$P=\left|\begin{array}{ccccc} p_{11} & p_{12} & p_{13} & p_{14} & p_{15}\\ p_{21} & p_{22} & p_{23} & p_{24} & p_{25}\\ p_{31} & p_{32} & p_{33} & p_{34} & p_{35}\\ p_{41} & p_{42} & p_{43} & p_{44} & p_{45}\\ p_{51} & p_{52} & p_{53} & p_{54} & p_{55} \end{array}\right|$$

Each probability $p_{ij}$ in equation (2) represents the probability of a child acquiring education level *j* when the parent’s education level is at level *i*. The direction of intergenerational mobility can also be seen when observing the magnitude of intergenerational mobility in education. In this paper, three main mobility indicators are calculated based on the transformation matrix: the solidification rate (*SR*), the upward mobility rate (*UR*) and the downward mobility rate (*DR*), as shown in Equation (3) to Equation (5). The solidification rate indicates the non-mobility ratio, which is the probability that the parent and the child are at the same educational level; the upward mobility rate indicates the probability that the child’s educational level is higher than the parent’s educational level; and the downward mobility rate indicates the probability that the child’s educational level is lower than the parent’s educational level. The downward mobility rate and upward mobility rate mainly reflect the direction of intergenerational mobility (Gu, 2012).


(3) $$SR=\underset{i=j}{\sum }a_{ij}/\sum a_{ij}$$

(4) $$UR=\underset{i>j}{\sum }a_{ij}/\sum a_{ij}$$

(5) $$DR=\underset{i<j}{\sum }a_{ij}/\sum a_{ij}$$

There are two main ways to build the mediating effect econometric model, one is to test the core variable coefficient to the mediating variable *a* and the mediating variable coefficient to the explained variable *b* sequentially, such as the sequential test coefficient method, and the other is to test the product of *a* and *b*, such as the Sobel test and Bootstrap test, etc. Since the derivation of the test statistic of the Sobel method requires the assumption that $\hat{ab}$ obeys a normal distribution, even if each of these coefficients is normally distributed, its product is usually not normal, thus the calculation of the standard error $S_{ab}$ above is only an approximation and may be inaccurate. Therefore, referring to the research method of Wen and Ye (2014), we try to use the one-time test coefficient method and Bootstrap test method for empirical testing, and the specific model is established as follows.


(6) $$Y=cX+w_{1}$$

(7) $$M=aX+w_{2}$$

….(8) $$Y=c^{\prime }X+bM+w_{3}$$

Coefficient *c* in equation (6) indicates the total effect of the explanatory variable *X* on the explanatory variable *Y*. Coefficient *a* in equation (7) indicates the regression coefficient of the explanatory variable *X* on the mediating variable *M*. Coefficient $c^{\prime }$ in equation (8) indicates the direct effect of the independent variable *X* on the dependent variable *Y* after controlling for the effect of the mediating variable *M*, and coefficient *b* is the effect of the mediating variable *M* on the dependent variable *Y* after controlling for the effect of the independent variable *X*. According to the test procedure of Wen and Ye (2014), the sequential regression test is conducted: firstly, the coefficient *c* of equation (6) is tested to make the explanatory variable regress on the explained variable; secondly, the coefficient *a* of equation (7) is tested to make the explanatory variable regress on the mediating variable; thirdly, the coefficient *b* of equation (8) is tested to make the explanatory and mediating variables regress on the explained variable at the same time. If the coefficient *c* is significant and both coefficients *a* and *b* are significant, the mediating effect is significant. Regarding the stepwise method of Baron and Kenny (1986), the first step tests the total effect of *X* on *Y*; the second step actually tests the significance of the product of the coefficients, indirectly by sequentially testing the coefficients *a* and *b*; and the third test is used to distinguish between full or partial mediation.

## 3. Results

### 3.1. Intergenerational transmission of education

Table 2 shows the overall transition between paternal and offspring education, from which it is clear that there is a positive relationship between parents’ education level and children’s education level. When parents’ education level is illiterate/semi-literate, the probability of children’s education level being high school or above is 24.85%, and the probability of children’s education level being university undergraduate or above is 3.49%; when parents’ education level is elementary school, the probability of children’s education level being high school or above is 42.43%, and the probability of children’s education level being university undergraduate or above is 6.24%; when parents’ highest education level is high school, the probability of children’s education level being high school or above is 69.38%, and the probability of children’s education level being university undergraduate or above is 6.24%. When the parent’s highest education is a bachelor’s degree, the probability that the child’s education is high school or higher is 85% and the probability that the child has a bachelor’s degree or higher is 40%. The solidification rate of intergenerational transmission of education between the father’s and children’s generations is 25.72%, upward mobility is 60.58% and downward mobility is 13.70%. The probability of upward mobility of children’s education is very high. It can be seen that if both parents’ educational attainment is at a low level, their children’s educational attainment is also low, and the probability of obtaining a university degree or above is very low, and when parents’ educational attainment increases, the probability of children’s educational attainment increasing increases, and when parents’ educational attainment is university or above, the probability of children’s educational attainment at junior high school or below is very low.

**Table 2:** Education intergenerational transmission transition matrix.

Highest parental education	Probability of children receiving the highest degree (%)							
	Illiterate / semi-literate	Primary school	Junior high school	Senior high school	College	Undergr-aduate	Master	PhD
Illiterate / semi-literate	11.42	21.55	42.17	16.76	4.60	3.13	0.18	0.18
Primary school	4.32	16.65	36.59	26.10	10.09	6.16	0.08	0.00
Junior high school	1.43	7.50	32.54	33.28	15.24	9.47	0.49	0.05
Senior high school	1.52	4.00	25.11	32.36	14.94	20.35	1.73	0.00
College	1.56	1.56	12.50	42.19	16.15	22.92	3.13	0.00
Undergraduate	1.00	1.00	13.00	37.00	8.00	34.00	6.00	0.00
Master	0.00	0.00	0.00	0.00	0.00	33.33	66.67	0.00

### 3.2. Children’s education in different family income classes

To investigate the relationship between family economic status and children’s educational attainment, family income quintiles are now divided and the samples within their quintiles represent families in five income classes: low-income families (0–20%), lower-middle-income families (20–40%), middle-income families (40–60%), upper-middle-income families (60–80%), and high-income families (80–100%). The household income profile and children’s education were linked to obtain Table 3.

**Table 3:** Distribution of children’s education under different family income.

Family village income quantile	Probability of children receiving the highest degree (%)							
	Illiterate / semi-literate	Primary school	Junior high school	Senior high school	College	Undergr-aduate	Master	PhD
0–20%	6.04	18.00	38.56	24.74	8.13	4.30	0.12	0.12
20–40%	3.91	12.08	36.23	29.19	10.25	7.73	0.52	0.09
40–60%	3.08	9.52	32.24	31.35	14.38	8.83	0.60	0.00
60–80%	2.47	6.53	31.75	32.44	13.95	11.97	0.89	0.00
80–100%	0.99	6.15	21.83	31.35	16.17	21.53	1.98	0.00

Table 3 shows that the higher the household income, the higher the educational attainment of the children of the household, especially the frequency of receiving education above bachelor’s degree will increase. The lower the family income, the lower the educational level of their children, especially the frequency of receiving education below junior high school will increase. Specifically, when the family income is in the low-income class, the frequency of their children’s education level being junior high school or below is 62.6%, the probability of being a bachelor’s degree or above is 4.54%, and the frequency of the children’s education level of low-income families is the largest being junior high school, 38.56%; when the family income is in the middle-income class, the probability of their children’s education level being junior high school or below is 44.84%, and the probability that they have a bachelor’s degree or above is 9.43%. When the household income is in the higher income bracket, the probability of having children with education level of junior high school or below is only 28.97%, and the probability of having children with university degree or above is 23.51%. This shows that the higher the family income level, the more likely their children will receive a higher level of education, especially higher quality education represented by a bachelor’s degree. For low-income families, the frequency of their children receiving undergraduate education is 4.30%, and for high-income families, this frequency rises to 21.53%, which is five times higher than for low-income families.

### 3.3. Mediating effects of household income on intergenerational transmission of education

Table 4 presents the results of the mediating effect of family income on the intergenerational transmission of education, with the regression coefficients and robust standard errors for each variable separately. In Table 4, Model 1 is the total effect of the effect of paternal education on the educational attainment of the offspring when no mediating variables are included, and the results show that at the 1% confidence level, paternal education has a significant positive effect on the educational attainment of the offspring, and a 1-year increase in paternal education is associated with a 0.279-year increase in the educational attainment of the offspring. The explanatory variable in model 2 is household income and the explanatory variable is paternal educational attainment, and the results show that at the 1% confidence level, a 1-year increase in paternal educational attainment increases household income by 7,220 CNY. This indicates that the educational attainment of the father’s generation has a positive effect on household income. Model 3 shows the results of the direct effect of paternal educational attainment on offspring’s educational attainment after the inclusion of the mediating variable, household income. Model 3 shows that at the 1% confidence level, paternal education still has a positive effect on offspring’s educational attainment, with an estimated coefficient of 0.272. The independent and mediating variables in models 1, 2, and 3 are significant, indicating a significant mediating effect. The effect of paternal education on offspring’s educational attainment was reduced by 0.007 with the inclusion of mediating variables, indicating that paternal education influences household income, which in turn has an effect on offspring’s educational attainment. Family income as a mediating variable explains 2.6% of the medium effect of paternal education on offspring’s educational attainment. Further testing of the model with Sobel-Goodman Mediation Tests revealed that all coefficients passed the test at the 1% significance level, providing further support for the mediating role of family income in the intergenerational transmission of education.

**Table 4:** Results of the mediating effect of household income on the intergenerational transmission of education.

Variable	Model 1	Model 1	Model 3
	ChildeduY	Fincome	ChildeduY
ParenteduY	0.279***	0.722***	0.272***
	(0.016)	(0.084)	(0.016)
Fincome			0.010***
			(0.003)
Health	−0.074	0.808***	−0.083
	(0.052)	(0.275)	(0.052)
Intelligence	0.173***	−0.583**	0.179***
	(0.049)	(0.259)	(0.049)
Family size	−0.159***	1.052***	−0.170***
	(0.029)	(0.154)	(0.029)
Intercept	8.965***	−1.234	8.978***
	(1.168)	(6.159)	(1.167)
Gender	YES	YES	YES
Province	YES	YES	YES
Urban	YES	YES	YES
Number of obs	3,610	3,610	3,610
*R* ^2^	0.192	0.102	0.195
Adj_R^2^	0.185	0.094	0.187
*F*	27.470	13.050	26.980

“*,” “**,” and “***” indicate that they are significant at the 10, 5, and 1% levels in turn, and are not significant if they are not marked. The numbers in parentheses in the table are standard errors.

## 4. Discussion

### 4.1. Robustness test

The Bootstrap method is a method of repeatedly sampling from a sample, provided that the sample is representative of the total. The Bootstrap method is also a direct test of the hypothesis H0: *ab* = 0. In this paper, the Bootstrap method is used to randomly sample 2000 times to test the robustness of the mediating effect of family financial investment in education between family income and children’s cognitive ability. The specific procedure is as follows: firstly, the original sample is repeatedly sampled 2000 times with put-backs to obtain 2000 Bootstrap samples; secondly, the estimates of the 2000 Bootstrap samples $\hat{ab}$ are obtained, and the whole estimates are expressed as { $\hat{ab}$ } ( $\hat{ab}$ is ordered by size), and the 2.5th percentile and 97.5th percentile of { $\hat{ab}$ } constitute the confidence level of *ab* is the 95% confidence interval; thirdly, if the confidence interval does not contain 0, then *ab* is significantly not equal to 0 and passes the mediating effect test. The results are shown in Table 5.

**Table 5:** Results of the test for mediating effects based on Bootstrap method.

	Observed coefficient	Bias	Bootstrap std. err.	95% Confidence interval		Percentile/ bias-corrected
				Lower limit	Upper limit	
Indirect effect	0.007	0.001	0.004	0.002	0.017	Percentile
				0.002	0.015	Bias-corrected
Direct effect	0.272	−0.001	0.017	0.238	0.307	Percentile
				0.239	0.308	Bias-corrected
Total effect	0.279	0.000	0.017	0.246	0.314	Percentile
				0.246	0.315	Bias-corrected

According to Table 5, the indirect effect of family income in the intergenerational transmission of education is 0.007, and the confidence interval of Bootstrap’s bias correction is [0.002, 0.015], this confidence interval does not include 0. This result indicates that the mediating role of family income in the intergenerational transmission of education holds, specifically, a 1-year increase in the parent’s educational attainment can indirectly increase the offspring’s education level by 0.007 years through the family income. Overall, the results of both the stepwise and Bootstrap methods indicate that the mediating effect is robust. Specifically, the total effect of paternal education on offspring’s education is 0.279, the direct effect is 0.272, and the mediating degree of family income is 2.6%. That is, 2.6% of the variation in offspring’s educational attainment is explained by the variation in household income due to parents’ educational attainment. This can indicate that higher education of parents has a positive effect on their household income and, in turn, higher education of their children.

To exclude the effect of variable selection on the model results, we re-estimated the mediating effect of household income on intergenerational transmission of education by replacing net household income with total household cash and savings (*deposit*) and total income of past respondents in the past 12 months (*fwage*) as mediating variables, respectively. The results of the resulting model are shown in Table 6.

**Table 6:** Results of the mediating effects model after replacing the main variables.

Variable	Model 4	Model 5	Model 6	Model 7
	Deposit	ChildeduY	fwage	ChildeduY
ParenteduY	0.514***	0.274***	0.415***	0.256***
	(0.075)	(0.016)	(0.031)	(0.016)
Deposit/fwage		0.011***		0.056***
		(0.004)		(0.009)
Health	−0.044	−0.074	0.330***	−0.090*
	(0.244)	(0.052)	(0.102)	(0.052)
IQ	0.115	0.172***	−0.054	0.172***
	(0.230)	(0.049)	(0.095)	(0.049)
Family size	0.295**	−0.162***	0.686***	−0.191***
	(0.136)	(0.029)	(0.057)	(0.030)
Intercept	3.875	8.923***	3.068	8.789***
	(5.466)	(1.167)	(2.255)	(1.161)
Gender	YES	YES	YES	YES
Province	YES	YES	YES	YES
Urban	YES	YES	YES	YES
Number of obs	3,610	3,610	3,546	3,546
*R* ^2^	0.081	0.194	0.235	0.200
Adj_R^2^	0.090	0.187	0.228	0.193
*F*	11.38	26.96	34.77	27.44

“*,” “**,” and “***” indicate that they are significant at the 10, 5, and 1% levels in turn, and are not significant if they are not marked. The numbers in parentheses in the table are standard errors.

According to Model 5, after using *deposit* as a mediating variable, the effect of parental education on offspring education decreases from 0.279 to 0.274, and the indirect effect is 0.006, which can explain about 2% of the total effect. According to Model 7, after using *fwage* as a mediating variable, the effect of paternal education on offspring education decreases from 0.279 to 0.256, and the indirect effect is 0.023, which can explain about 8% of the total effect. The results in Table 6 indicate that family income plays a significant mediating role in intergenerational educational transmission and that differences in family income are a non-negligible factor in differences in educational attainment.

### 4.2. Other influencing factors

It is generally accepted that an individual’s educational achievement is influenced by three factors: individual giftedness, which is determined by genetic inheritance; family investment in education (Leibowitz, 1974), which is determined by family income and preferences for children; and public investment in education, which is determined by government spending on education (Becker and Nigel, 1979). This paper covers giftedness and family income in its model, but the effect of public education investment on the intergenerational transmission of education has not been discussed. Among the implemented policies, the expansion of colleges and universities in China is the most influential and widest-ranging policy. The following discussion focuses on the impact of the college expansion policy on the mediating effect of family income in the intergenerational transmission of education. Since the expansion of colleges and universities in China took place after 1999, it mainly affects the offspring born after 1981. In this paper, we divide the sample into two groups: those born before 1981 and those born after 1981, and analyze the impact of the college expansion policy by comparing them. The obtained results are shown in Table 7.

**Table 7:** Results of the mediating effect of college expansion on household income.

Variable	Model 8	Model 9	Model 10	Model 11	Model 12	Model 13
	ChildeduY	Fincome	ChildeduY	ChildeduY	Fincome	ChildeduY
ParenteduY	0.297***	1.593**	0.258***	0.274***	0.650***	0.268***
	0.077	0.622	0.079	0.016	0.084	0.017
Fincome			0.025*			0.009***
			0.013			0.003
Health	−0.064	1.510	−0.101	−0.073	0.622**	−0.079
	0.315	2.535	0.311	0.053	0.269	0.053
IQ	0.255	−1.670	0.296	0.169***	−0.399	0.173***
	0.304	2.450	0.300	0.050	0.254	0.050
Family size	−0.037	−0.794	−0.017	−0.159***	1.106***	−0.169***
	0.148	1.190	0.146	0.030	0.152	0.030
Intercept	9.723***	−10.488	9.983***	8.637***	−0.496	8.642***
	3.353	26.994	3.306	1.246	6.329	1.244
Gender	YES	YES	YES	YES	YES	YES
Province	YES	YES	YES	YES	YES	YES
Urban	YES	YES	YES	YES	YES	YES
Number of obs	126	126	126	3,484	3,484	3,484
*R* ^2^	0.479	0.515	0.500	0.191	0.106	0.192
Adj_R^2^	0.315	0.362	0.335	0.183	0.098	0.185
*F*	2.91	3.36	3.03	26.20	13.21	25.67

“*,” “**,” and “***” indicate that they are significant at the 10, 5, and 1% levels in turn, and are not significant if they are not marked. The numbers in parentheses in the table are standard errors.

According to Model 8, the coefficient of the effect of paternal education on the offspring’s education before the expansion of higher education is 0.297, and according to Model 10, the indirect effect of family income is 0.039, which explains about 13.2% of the effect of paternal education on the offspring’s education. According to Model 11, after the expansion of colleges and universities, the coefficient of parental education on offspring’s education is 0.274, and according to Model 13, the indirect effect of family income is 0.006, which explains about 2.2% of the effect of parental education on offspring’s education, which is 11% lower than before the expansion of colleges and universities. In 1999, college enrollment increased by 513,200, reaching a total enrollment of 1,596,800, with a growth rate of 47.4%, and the expansion rate was 38.16% in 2000, 21.61% in 2001, and 19.46% in 2002. By 2003, the number of undergraduate and college students in China’s general universities exceeded 10 million. With no significant increase in college tuition, the weakening effect of college expansion on family income in the intergenerational transmission of education is very obvious.

## 5. Conclusion

This paper selects relevant data from CFPS 2018 and examines the intergenerational transmission of education in China, the mediating effect of family income in the intergenerational transmission of education and the effect of college expansion on the mediating effect of family income in the intergenerational transmission of education through the transformation matrix as well as the test of mediating effect. Based on the previous empirical study, the main findings of this paper are as follows.

1. The educational attainment of parents has a significant transmission effect on the educational attainment of children, with a solidification rate of 25.72%, an upward mobility rate of 60.58% and a downward mobility rate of 13.70% for the intergenerational transmission of education between the father’s and children’s generations. When both parents’ educational attainment is at a low level, the probability of their children obtaining a college degree or higher is very low, and when parents’ educational attainment increases, their children’s educational attainment will increase accordingly. The probability of children of parents who have received a college degree or higher education is significantly higher than that of others.
2. The mediating effect model shows that the total effect of the parents’ educational attainment on the offspring’s educational attainment is 0.279 and the direct effect is 0.272, and the family income plays a mediating effect in the intergenerational transmission of education, with the degree of mediation reaching about 2.6%, that is, 2.6% of the difference in the offspring’s educational attainment is explained by the difference in the family income caused by the parents’ educational attainment, and it can be said that the higher the parents’ educational attainment, the higher the positive effect on their family income. The higher the educational attainment of the parents, the higher the positive effect on their household income and, in turn, the higher the educational attainment of the children.
3. The expansion of colleges and universities provides more opportunities for children of society, especially those from lower and middle-income families, to receive higher education, weakening the mediating effect of family income in the intergenerational transmission of education. After the expansion of higher education, the mediating effect of family income in the intergenerational transmission of education decreases from 13.2 to 2.2%.

The limitation of this paper is that it does not further analyze the mechanism of the influence of family income on the intergenerational transmission of education. Future research can provide insight into the pathways of family income influence in the intergenerational transmission of education in terms of families’ financial investment in education, parents’ educational expectations, and parents’ involvement in education. Compared with previous studies, the contribution of this paper is to empirically test the effect of household income on intergenerational transmission of education using a mediating effects model and to provide statistical evidence for this effect based on Chinese household surveys. The conclusions of this paper also provide guidance for policy makers to further strengthen public investment in education.

## Data availability statement

The datasets presented in this study can be found in online repositories. The names of the repository/repositories and accession number(s) can be found in the article.

## Author contributions

XL: Conceptualization, methodology. WX: Supervision, review and editing. LW: writing–review and editing. TZ: analysis and discussions. All authors contributed to the article and approved the submitted version.

## Funding

This research was financially supported by the National Social Science Foundation of China (17CRK009).

## Conflict of interest

The authors declare that the research was conducted in the absence of any commercial or financial relationships that could be construed as a potential conflict of interest.

## Publisher’s note

All claims expressed in this article are solely those of the authors and do not necessarily represent those of their affiliated organizations, or those of the publisher, the editors and the reviewers. Any product that may be evaluated in this article, or claim that may be made by its manufacturer, is not guaranteed or endorsed by the publisher.
